# World mental health on a tightrope: evidence, practice, policy and *BJPsych International*

**DOI:** 10.1192/bji.2024.6

**Published:** 2024-05

**Authors:** Marinos Kyriakopoulos

**Affiliations:** Assistant Professor in Child and Adolescent Psychiatry, School of Medicine, National and Kapodistrian University of Athens, Athens, Greece. Email: mkyriakop@med.uoa.gr

Taking over from David Skuse as editor of *BJPsych International* is both a privilege and a big task. David has steered the journal into the digital age, transformed its content, broadened its reach and expanded its online presence. He oversaw the journal being indexed in PubMed, which has been a milestone contributing to its growth. In the past decade, under David's leadership, *BJPsych International* has gone from strength to strength and developed as a widely accessible platform for best practice and policy in mental health. I have learned a lot from David, from my first steps in 2012 as a member of the editorial board of the journal (then called *International Psychiatry*) to associate and deputy editorships later. I am very pleased he has agreed to remain on our editorial board alongside the group of amazing expert colleagues who supported *BJPsych International* in different roles over the years. Within the *BJPsych* publications family, our journal has a unique part to play in sharing, promoting and shaping excellence in mental health practice across all countries and cultural contexts.

## Mental health around the world

Mental health is increasingly being recognised as a major public health priority. Mental disorders are highly prevalent worldwide and constitute the leading cause of years lived with disability, as well as being major drivers of excess morbidity and mortality.^1^ The economic costs associated with them, both directly and indirectly, are also exceptionally high. Despite this, underfunding of services and commissioning of suboptimal services are the rule rather than the exception. Several global initiatives, most notably the Comprehensive Mental Health Action Plan 2013–2030 by the World Health Organization, aspire to address this through forming systems where promotion of mental health, prevention of and recovery from mental disorders, and development of high-quality culturally appropriate interventions are enabled.^2^ Effective leadership and universal, comprehensive, multisectoral, integrated and responsive evidence-based services are paramount for this aim to be achieved. This cannot materialise without the involvement of patients and commitment to safeguarding and respect of human rights every step of the way, from research to policy implementation.

However, despite some positive steps in this direction, there is still a considerable distance to go. Mental health systems in most countries are lacking the governance and resources to provide for all those in need, resulting in major gaps in service quality and coverage. Competing demands with physical healthcare often find mental healthcare disadvantaged. On average, countries dedicate less than 2% of their healthcare budgets to mental health, with related practices being more affected in low- and middle-income countries. More than 70% of health funds support psychiatric hospitals in middle-income countries, whereas in low-income countries, not enough psychiatrists or psychotropic medicines are available.^1^ Poor mental health literacy and associated stigma are further barriers to accessing existing services. Research from low- and middle-income countries is scarce, which is an impediment to the advancement of a culturally relevant evidence base and driving policy.

## Mission and aims of *BJPsych International*

In this context, *BJPsych International* strives to bridge a knowledge gap by providing an overview of current policy and practice in psychiatry – and, more widely, mental health – in countries around the world and fostering the implementation of evidence-based approaches. This is facilitated by an editorial board of academics and senior clinicians from all continents, and through its range of content, including different types of article. Special Papers and Thematic Papers deal with the policy and promotion of mental health, the administration and management of mental health services, worldwide training in psychiatry, and knowledge and best practice from low- and middle-income countries through examination of the relationships among culture, mental health and well-being. Country Profiles provide summary information on mental health policy, services, training and research in a specific country. Mental Health Law Profiles summarise national or regional mental health law, including recent developments, and give commentaries on its application and the monitoring of standards in practice. Editorials provide an expert introduction, or an informed summary of published articles. Global Echoes focus on international mental health work and include brief literature reviews on mental health policy or services, reports of elective projects in psychiatry, or other experiences or challenges of working or volunteering in different countries. Finally, Pandora's Box highlights research findings, news and matters of interest from around the world. Most of these article types are relatively brief, typically up to 1500 words with up to 12 references. In addition to printed content, *BJPsych International* has increased its online visibility through the creation of videos and podcasts (https://www.cambridge.org/core/journals/bjpsych-international/videos-and-podcasts#videos) and its popular arts blog *Muses* (https://www.cambridge.org/core/blog/tag/muses-the-arts-blog/).

The time has now come for *BJPsych International* to expand into publishing original research and systematic reviews and meta-analyses, directly contributing to building an evidence base for good practice and policy worldwide, with a special focus on countries that have historically contributed little to the international scientific literature. It has been recognised that a significant increase in the word count for these papers would be necessary in order for the journal to attract top-quality submissions. To that end, two new article types will be introduced: Original Articles, up to 4000 words with up to 40 essential references; and Reviews, up to 6000 words with up to 150 essential references. Together with the fact that *BJPsych International* is open access, not charging a publication fee to the authors of accepted papers or to its readers, this will further elevate the journal to a key publication in the field of international psychiatry and mental health, facilitating cross-fertilisation without barriers across all cultures and continents.

## Summary

Capitalising on its strengths, *BJPsych International* continues its exciting journey of growth and development. With a prominent online presence, a large readership, clinically relevant content and a high commitment to making a difference, the journal is placed in an excellent position to serve its mission. The inclusion of original research and systematic reviews and meta-analyses will further advance its impact; we welcome submissions of such papers from around the world!
